# The Cognitive-Affective-Social Theory of Learning in digital Environments (CASTLE)

**DOI:** 10.1007/s10648-021-09626-5

**Published:** 2021-06-30

**Authors:** Sascha Schneider, Maik Beege, Steve Nebel, Lenka Schnaubert, Günter Daniel Rey

**Affiliations:** 1 Psychology of Learning with Digital Media, Faculty of Humanities, Chemnitz University of Technology, Straße der Nationen 12, 09111 Chemnitz, Germany; 2 Fachgebiet Psychologische Forschungsmethoden - Medienbasierte Wissenskonstruktion, Universität Duisburg-Essen, Campus Duisburg, Lotharstraße 65, 47057 Duisburg, Germany

**Keywords:** Social cues, Digital environments, Multimedia learning, Cognitive processing

## Abstract

For a long time, research on individuals learning in digital environments was primarily based on cognitive-oriented theories. This paper aims at providing evidence that social processes affect individual learning with digital materials. Based on these theories and empirical results, a social-processes-augmented theory is suggested: the Cognitive-Affective-Social Theory of Learning in digital Environments (CASTLE). This CASTLE postulates that social cues in digital materials activate social schemata in learners leading to enhanced (para-)social, motivational, emotional, and metacognitive processes. To substantiate this theory, socio-cognitive theories are used, which predict social influences on learning with digital materials. Besides, previous empirical findings are presented assuming that with a rising number of social cues in digital materials, the influence of social processes increases. Finally, consequences regarding the design of digital learning media are discussed.

## Introduction

The proliferation and rapid progress of digital learning technologies require educational designers and teachers to continuously create or choose digital learning materials. This is even more relevant since especially young learner’s digital media use has considerably increased over the past 10 years. For example, research has found teenagers gathering information online and visiting social media sites for more than 6 hours a day (Twenge et al. 2019). Digital learning material is particularly characterized by seamless learning, which is defined as a “seamless flow of learning across contexts” (Wong and Looi 2011, p. 5) shifting the learning process towards a much more personal experience. Some theories and recent research findings predict that the learning process with digital materials is still perceived as a social interaction process (e.g., Apps et al. 2019; Liew et al. 2020). These social processes are elicited by social cues (Mayer et al. 2003), which are defined as all material-based traces of humans including all phenomena attributing human characteristics to digital learning material. It is of central importance that the influence of social processes when learning with digital materials should be investigated in closer detail to be able to predict the influence of social processes on learning in future studies. This paper reviews general theories on how individuals might experience social processes in learning environments to propose specific hypotheses on social processes in digital learning environments. In addition, a theory on social processes in digital learning environments is outlined. Its hypotheses are further defined by specific theories and recent experimental studies on social processes when cognitively processing information.

## Learning in Digital Environments

The COVID-19 crisis has made schools and other educational institutions more aware than before of how important digital learning materials can be for the successful continuation of learning. Learning texts and illustrations are being exchanged digitally, both self-made and externally produced educational videos sent. The range of digital learning environments is wide. Learning in digital learning environments is discussed in this paper as an interaction of one learner with digital learning material. Digital learning materials are defined here in the broadest sense from basic text-image combinations to dynamic media, that is, media that illustrates information time-dependent, such as videos or animations, and to interactive media, that is, media that allows for intervention, control, and feedback possibilities, such as simulations, educational video games, or online tests. Since primarily individual learners are in such a digital environment, this review and the proposed theory only refer to an instructional interaction that is designed as learning or retrieving task for one learner, therefore excluding truly collaborative learning scenarios involving direct interaction of more than one learner.

### Cognitive Theories on Learning with Digital Materials

For the past 40 years of digital learning research, learning was mainly described by cognitive-oriented learning theories. According to the cognitive load theory (CLT; Sweller 1988, 2020; Sweller et al. 1998, 2019), information within digital learning material is processed during a learning process within the working memory system to transfer it into long-term memory. Consequently, learning is defined as a change in the long-term memory system, which is assumed to be unlimited in its capacity. However, the working memory system is found to be limited in its capacity. This limitation leads to the fact that processed information can be defined as a load for learners. Learners should therefore not be overloaded during their learning process so that all information relevant to learning can be processed, organized into mental models or schemata, and transferred into long-term memory. Processing learning-relevant information causes a learning-related cognitive load (i.e., intrinsic cognitive load; ICL). In addition, the intrinsic cognitive load is moderated by three factors: (1) the level of element interactivity, defined as the number of elements that have to be processed simultaneously in the working memory system; (2) the amount of domain-specific prior knowledge of learners, defined as the knowledge in the specific learning domain; and (3) the degree to which learners have to build and automatize new mental models (i.e., germane cognitive load). A high intrinsic cognitive load is therefore present when learners face a high element interactivity, do not have any domain-specific prior knowledge, and thus have to build and automatize new mental models. Depending on the type of presentation or design of the learning environment, information in a digital learning material can be easier or more difficult to process—changing the amount of learning-irrelevant cognitive load (i.e., extraneous cognitive load). In consequence, instructional designers aim to reduce extraneous cognitive load to enable learners to cope with intrinsic cognitive load processes.

The cognitive theory of multimedia learning (CTML; Mayer 2014a) represents another central theory of learning with digital media besides the CLT. This theory provides a framework model of the entire learning process and is based on three assumptions: (1) Information is processed via two cognitive channels, an assumption which is based on theories of Paivio (1986) and Baddeley (1992); (2) based on the working memory model by Baddeley (1992), the working memory system is limited in its capacity; and (3) learners need to actively process information to build coherent mental representations and models. Moreover, the CTML distinguishes between five different cognitive processes that can occur during learning with digital materials (Mayer and Moreno 2003). These include the selection of relevant words (1) and images (2), the organization of the selected words (3) and images (4), and the integration of the verbal and pictorial mental model with the learner’s prior knowledge (5; i.e., the building of a coherent mental model). Typically, according to Mayer (2014a), these cognitive processes take place again for each section within the multimedia message.

### Previous Extensions of Cognitive Learning Theories

To date, there are some extensions of these cognitive theories on learning with media which put a focus on, for example, additional metacognitive (i.e., holding information about cognition and internal states as well as coping strategies that impact both; Wells and Capobianco 2020) and/or affective (i.e., a combination of motivational and emotional) processes.

The augmented cognitive load theory (aCLT; Huk and Ludwigs 2009) assumes, for example, that emotional and cognitive processes can be intertwined when both are elicited by a learning material. The integrated cognitive affective model of learning with multimedia (ICALM; Plass and Kaplan 2015) described this intertwining by using the CTML framework. The CATLM (i.e., the cognitive-affective theory of learning with media; e.g., Moreno 2006) is also based on the three basic CTML assumptions but also makes four extended basic assumptions that describe the factors that mediate or moderate the learning process: (1) the affective mediation hypothesis, which assumes that affective and motivational factors mediate the learning process; (2) the metacognitive mediation hypothesis, which assumes that metacognitive processes also influence the learning process; (3) the assumption that the long-term memory has to be divided into a semantic and an episodic part; and (4) the individual difference hypothesis, which describes that the learning process is additionally influenced by individual differences in learners. For example, previous knowledge or individual learning styles are possible moderators.

Overall, the extension of cognitive learning theories stresses that a learning process is not solely based on cognitive processing of information but also emotional, motivational, and metacognitive processes. Learning with digital learning materials, however, can also trigger social processes, such as the feeling that the interaction between a learner and a digital device is rather social than technical. For this, the following sections will not only inform on how social processes arise in digital environments but also how these theoretical consideration result in a direct derivation of hypotheses.

## Fundamental Theories on Social Processes in Digital Environments

### The Computers-As-Social-Actors Paradigm and the Media Equation Theory

One main concept that explains the relationship between digital environments and individual human beings as social interactions is the computers-as-social-actors paradigm (CASA paradigm; Nass et al. 1994). According to this theory, computer-based interaction can be primarily interpreted as a social event, while social cues are the basis of this event (Moreno and Mayer 2000; Reeves and Nass 1996). Social cues within media environments (e.g., voice, eye gaze, or gestures) prime a social activation schema. In consequence, social processes and scripts of human-to-human communication are triggered, as in the cooperation principle (Grice 1975; see section “Social Representations, Social Schemata, and Collaboration Scripts”). This applies to conventional computers as well as newer technologies like smartphones (Carolus et al. 2019). Despite the change in knowledge and experience of media users, the advance of technology, and the change of human-computer interaction (Gambino et al. 2020), current research implies that humans, for example, a learner who gains knowledge from a multimedia learning environment, can interpret the interaction with technology as a social event even if no other actual humans are present (Xu and Lombard 2016).

The CASA paradigm is derived from the media equation theory (Reeves and Nass 1996), which states that even if humans do not explicitly attribute human-like characteristics to technology, they are behaving socially towards computers or robots (Nass and Moon 2000). These social reactions are driven by implicit anthropomorphism, the tendency to attribute mental characteristics to non-human objects (Waytz et al. 2013), which is easy to generate. Simple cues such as voices or personifications within a multimedia environment create the sense of a social presence (the awareness of another social entity during a communication process; Lee and Nass 2003) and lead to social responses. Cues of humanness support cognitive processes and encourage people to apply social rules (Nass and Moon 2000). Whereas the classical media equation theory pointed out that simple cues are sufficient for a social response from humans, recent research focusses on the implementation of additional and more complex cues to trigger more specific responses (Klowait 2018). In sum, the CASA paradigm supports the claim that multimedia environments can trigger social processes even if there is no real group situation with other people present. Thus, individual learning can be influenced by social cues in digital technologies leading to the following hypothesis:

*Activation Hypothesis: Social cues activate social schemata and trigger social processes.*

Actual empirical studies pointed out that these processes are not fully implicit and automated: The media equation effects depend on individual differences (Fischer 2011) as well as anthropomorphic features of the environment (e.g., emotional expression; Złotowski et al. 2018). These cues leading to a perception of a social interaction with such media are further described by the concept of social presence.

### Social Presence

According to the social presence theory (Short et al. 1976), all media which cause a user’s perception of a person being present can be defined as media with social presence (Dunlap and Lowenthal 2014; see also Bickle et al. 2019). This means that users can identify groups within media, communicate openly in a trusting digital environment, and develop relationships by projecting individual personalities within media settings (Garrison 2016). If a person is acknowledged as “real” depends on the intimacy and immediacy of a medium, which are communicated through the verbal and nonverbal responses of a digital environment (e.g., Argyle and Dean 1965; Biocca et al. 2003; Cui et al. 2013), such as immediate and intense as eye contact with agents, proximity, or response time. In consequence, humans can perceive social presence during individual media reception in dependence on media design. This applies to casual media reception as well as to individual learning scenarios using digital technologies. While face-to-face communication and synchronous video-mediated digital materials can be classified as high in social presence, text-based communication is low in social presence (Whiteside et al. 2017). The fact that the type of media has an influence on how strong social cues active social schemata (i.e., values, ideas, beliefs, and practices that are shared among members of a group or even whole communities; Moscovici 1981) leads to the following hypothesis:

*Social Cue Strength Hypothesis: The strength of social cues to activate social schemata is moderated by the modality of the learning material, the temporal changeability of information, as well as the degree of interactivity of a learning material.*

When interacting with media displaying media persons, not only perceptions of social presence but also parasocial interaction processes need to be considered.

### Parasocial Interaction

Parasocial interaction (PSI) describes the manifold influences of media persons (persona; Hartmann et al. 2004) on media recipients and goes back to basic research in social psychology (Horton and Wohl 1956). PSI is defined as a process that changes dynamically in the process of media reception (Cummins and Cui 2014; Tsay-Vogel and Schwartz 2014) and can thus have a beneficial effect on cognitive processes relevant to individual learning. This can be seen from the two-level model of parasocial interaction (Hartmann et al. 2004).

According to this PSI theory, media persons influence the media recipient on a cognitive, affective, and behavioral level depending on their appearance and presentation, but there is no communicative return channel of the recipient to the persona (Hartmann et al. 2004). Furthermore, it becomes clear that both characteristics of the media recipient and characteristics and representations of the persona affect the intensity of the PSI. The cognitive facet comprises attention and evaluation processes, activation processes of prior knowledge and experience, as well as anticipation and understanding processes. According to this theory, elicited social and parasocial processes thus influence the cognitive processing of new information leading to the following hypothesis:

*Cognitive Influence Hypothesis: (Para-)Social processes influence how information is selected, organized, integrated, and retrieved.*

Affective elements include sympathy, antipathy, empathy, and processes of emotional transmission. The behavior-oriented component includes nonverbal behavior, (para-)verbal behavior, and behavioral intentions. Due to the dynamic representation of the persona, PSI and its facets are subject to constant change (Schramm and Wirth 2010). Studies have shown that strong PSI processes lead to higher engagement and enjoyment in digital technologies (Tsay-Vogel and Schwartz 2014), stronger identification with media characters (even with amoral personas; Oliver et al. 2019), while PSI is also correlated with persuasiveness (Rosaen et al. 2019). Considering these multiple influences of parasocial processes on individual learners, the theory of PSI should be considered when discussing educational processes in digital environments.

Both social and parasocial processes are thus not only found to affect cognitive, but also metacognitive, emotional, and motivational processes and vice versa (e.g., Homer et al. 2008; Fiz-Pérez et al. 2016; Park 2015; Schramm and Wirth 2010) leading to the following hypothesis:

*Interaction Hypothesis: Social processes influence and are influenced by motivational, emotional, and metacognitive processes.*

However, the activation of social and parasocial processes not only leads to the unconscious adaptation of cognitive processes, but also to a process of comparison that leads to active monitoring and control of the learning processes.

### Social Comparison Processes

The *Theory of Social Comparison Processes* by Leon Festinger (1954) is a fundamental framework when it comes to a comparison between humans and other (artificial) social entities. Primarily, it is postulated that humans inherit a drive to evaluate their opinions and abilities (i.e., gathering feedback; see section Feedback and Social Processes), and if no objective standard exists, they will use any available entity. Within a digital learning setting, it can be concluded that if no standard has been explicitly established by the instructor, the learner will try to evaluate his/her ability with any available social or even parasocial entity. In addition, it is irrelevant whether the included social cues are based on real human beings or are generated completely artificially. Because of this, the theory was successfully applied to individual learning where a social component was substituted with simpler social cues (e.g., a leaderboard; Nebel et al. 2017a).

The processes triggered through comparisons with the artificial or simulated social entity can be understood as a feedback process, as learners try to infer an understanding of their abilities in relation to other various (artificial) information sources (e.g., Kollöffel and de Jong 2016; Neugebauer et al. 2016). Depending on the outcome, different results might occur (e.g., positive feedback, negative feedback). Thus, this mechanism could be controlled and optimized regarding the performance of learners (e.g., intelligent tutorial systems), whereas real social interactions induce a level of unpredictability. Secondly, Festinger (1954) postulated that the strength of this (para-)social interaction and presumably the impact on learning processes depend on several factors, such as the similarity of skills and abilities between the social entities or the individual relatedness. Comparisons within highly relevant areas, close peers, or abstract representations of them (e.g., a leaderboard indicating that my virtual best friend performed better; e.g., Nebel et al., 2020) might strengthen the impact of the inferred feedback, while irrelevant topics and highly different comparison partners (e.g., a teacher-like avatar whom I do not like is presented to be better at a topic that is not of interest to me) might reduce it substantially.

Besides, researchers explored how the number of comparison partners might influence the comparison process (Garcia and Tor 2007, 2009; Garcia et al. 2006). As a consequence, different impacts within small-scaled instruction, such as individual multimedia learning and larger groups, such as virtual classrooms, could be expected. Festinger (1954) assumes a motivational and a behavioral component. If a discrepancy in abilities is detected, the person will act to reduce them. In the case of abilities, humans inherit a unidirectional drive upwards resulting in behavior trying to improve the compared abilities. However, it has to be noted that some behavioral outcomes of the social comparison process might be diametrical to learning. For instance, stronger comparisons within homogenous groups can be derived from Festinger’s (1954) framework, whereas heterogeneity might be more common and even beneficial for the learning process (e.g., providing a wider range of opinions, ideas, and skillsets). Comparisons, however, might also lead to conflict (see section “Conflict Elaboration Theory and Socio-cognitive Conflicts”). Also, the upwards-driven behavioral result of this comparison can lead to actions protecting one’s superiority and, as a consequence, inducing learning-hindering effects for the remaining social group (e.g., hoarding knowledge; Ray et al. 2013). Overall, this framework supports the inclusion of social comparison processes within the examination of individual learning processes with digital materials as soon as any form of social cue triggers self-evaluation. However, the strength and speed of the activation of social schemata depend on various factors, such as the number of social cues (e.g., Garcia and Tor 2009), the strength of social cues (e.g., Nebel et al., 2020), as well as the degree of social schema development (e.g., Fischer et al. 2013; Schnaubert et al. 2020b) leading to the following hypothesis:

*Schema Influence Hypothesis: How strong and fast social schemata are activated depends on the strength and number of the social cue as well as the degree of development of the social schemata.*

### The Need for a Multimedia Learning Theory Including Social Processes

The fundamental theories on social processes in digital learning materials presented so far highlight the importance of information eliciting social processes within digital learning environments. In research on learning in digital environments, however, there is, so far, no theory that addresses the social impact of digital materials on learning processes. One theory that extended the learning process with multimedia learning materials the most can be seen in the CATLM. Nonetheless, this theory does not include how social processes affect these learning processes. For this, the present paper was aimed to create a theory on learning with digital learning materials, which also includes the notion that all interactions between a human and a technical device-like computers or machines have the potential to be social interactions (cf. CASA paradigm). Based on the plurality of technical devices and the instructional message as well as individual differences in humans, this potential can be activated or not. The probability of activating such a potential, however, can be raised by including social cues into the digital learning material.

## The Cognitive-Affective-Social Theory on (digital) Learning Environments (CASTLE)

**Fig. 1:**
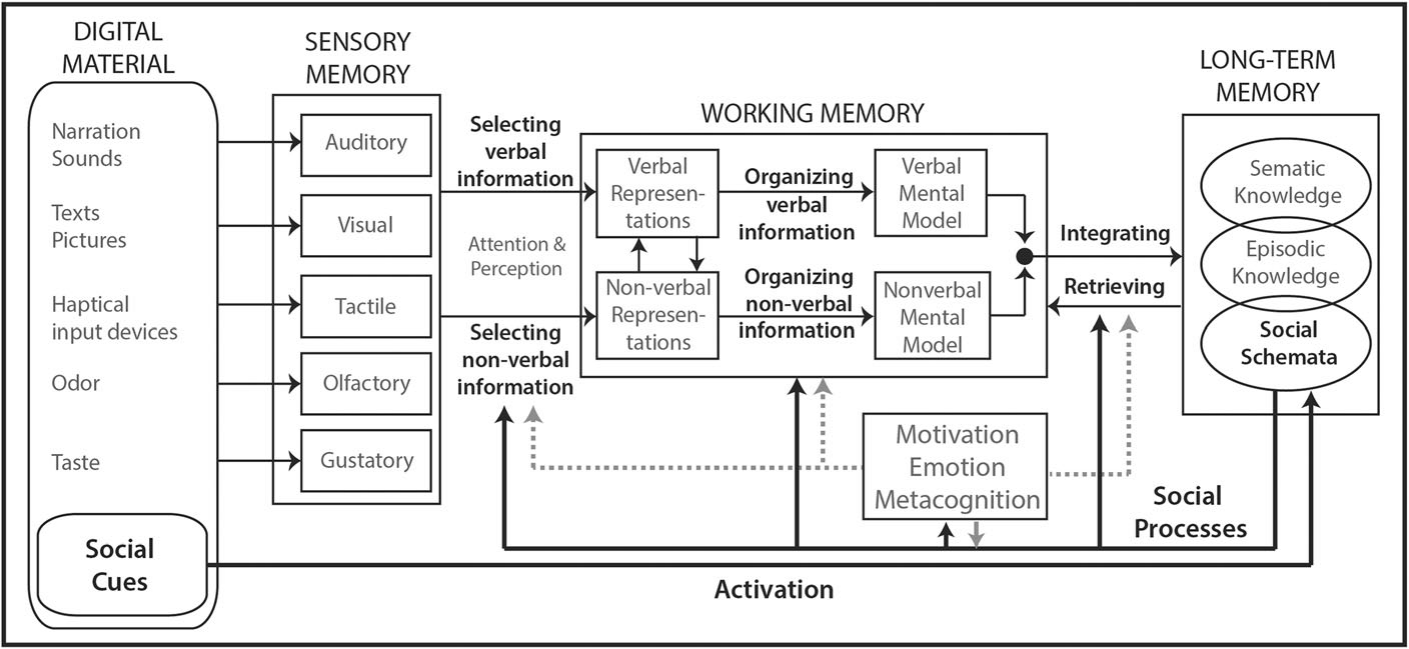
The Cognitive-Affective-Social Theory of Learning in digital Environments (CASTLE) with emphasis on the mediation of social processes on selection, organization, integration, and retrieval processes. The CASTLE is based on the Cognitive-Affective Theory of Learning with Media according to Moreno (2006)

The preceding models of social processes provide evidence that social processes occur in the interaction between humans and instructional messages mediated by technical devices. However, this assumption is not yet included in any theory or framework on learning with digital materials. Based on the basic assumptions of the CTML and the CATLM, this paper adds another basic assumption—the social mediation hypothesis: *Social processes triggered by social cues mediate the cognitive processing of information when learning with digital materials.* This hypothesis postulates that both social and parasocial processes occur in the reception of digitally presented learning materials and thus influence learning. This basic assumption was used to build a new theory: the Cognitive-Affective-Social Theory on (digital) Learning Environments (CASTLE; see Fig. 1).

In detail, the basic assumption of the CASTLE can be divided into several sub-hypotheses: First, social cues, which are shown to be inherently present in digital learning environments, trigger activation of social schemata in the long-term memory of learners (see Activation Hypothesis). Second, this activation leads to social and parasocial processes influencing all cognitive processes involved in learning: the selection of information, the transfer of information into working memory through attention processes, the organization of information into mental representations or models, the integration of mental models into the long-term memory system, as well as the retrievability of stored information (see Cognitive Influence Hypothesis). Third, social processes are by no means to be regarded separately from other mediating factors. Social processes can influence or be influenced by affective, motivational, and/or metacognitive processes (see Interaction Hypothesis). To illustrate this, the CATLM model was extended by this social mediation hypothesis to develop the Cognitive-Affective-Social Theory of digital Learning Environments (CASTLE; for an overview, see Fig. 1).

Fourth, the degree of activation of social schemata through social cues is postulated to increase with the number and strength (e.g., salience) of the social cues provided in a digital learning environment, but also with the level of the development of the social schema (with more developed social schemata being easier to activate; see Schema Influence Hypothesis). Fifth, while learning materials with verbal instructional messages only, such as learning texts, have fewer opportunities to inherit social cues, visual stimuli, such as illustrated learning texts, rely on more possibilities to trigger social processes. Moreover, if digital learning materials are dynamic or even interactive, social traces increase, and thus, the social schema activation potential is supposed to raise (see Social Cue Strength Hypothesis). In order to strengthen the main hypothesis and sub-hypotheses of the CASTLE, further relevant and empirically validated theories and empirical results are described refining the processes activated by social cues.

## Combining Cognitive and Social Processes during Learning in Digital Environments

### Social Constructivist Theory and Social Cognitive Theory

One of the earliest learning theories combining learning science and social psychology is the social constructivist theory (Vygotsky 1978) assuming that knowledge is mainly formed in social interactions and a specific socio-cultural environment. Thus, this theory is of great importance for the CASTLE. Through language, people in social interactions exchange purposes and extract meaning. Based on this theory, learning can be defined as all social interactions leading to the acquirement of experience (Vygotsky and Kozulin 1989). However, not all interactions between humans and computers can be defined as inherently social. In this vein, the s*ocial cognitive theory* (Bandura 1986) claims that effective learning cannot only take place in social interactions but also through modeling or observations represented in situations where individuals learn with digital materials. In the case of digital learning environments, humans can be seen as agents of the successful acquisition of learning. By observing people acting or, in a wider sense, by acquiring knowledge, which is based on people’s experiences, learners’ cognitive, emotional, motivational, and metacognitive processes are influenced. According to the triadic reciprocal causation of Bandura (2001), it needs three factors for an optimal learning process: person, behavior, and environment. Each factor can influence the other. For example, environmental factors, such as the general mood portrayed in learning material, can affect a person by changing his or her interest or his or her cognitive engagement (i.e., behavior). Based on Bandura’s theory, digital learning environments can function as this environmental factor influencing learning processes affecting learners.

### Socially Situated Cognition

According to the socially situated cognition theory (e.g., Smith and Semin 2004), learning cannot be studied by focusing only on the isolated cognitive functions, such as attention and perception, mental model building, or memorization, but on the interplay between own behavior, the behavior of others, and environmental resources (Semin et al. 2012). “Social” in this sense is described as an adaptive co-regulation of action between interaction partners (e.g., Semin and Cacioppo 2008). Supporting the CASTLE, such an interaction partner can also be found in digital learning environments, whereby the regulation is often one-sided. Socially situated cognition is more connected with mapping knowledge to the perceptuomotor repertoire of, for example, media users (Buccino et al. 2004), which is, in turn, a connection between knowledge and a personal perspective. Both the mental model and the interaction with the environmental resource can lead to the activation of knowledge (i.e., the retrieval process). In addition, the more social interaction such an environment offers (e.g., interacting with pedagogical agents), the more connections can be used to retrieve knowledge.

### Social Facilitation and Social Inhibition

Based on the social presence theory (described in section “Social Presence”), the CASTLE postulates that an increased perception of social presence can lead to an increased task performance in multimedia learning environments—a phenomenon called social facilitation (Triplett 1898; for a review see Seitchik et al. 2017)—an effect that is also biologically verified (Demolliens et al. 2017). For example, students solving a stroop task (i.e., naming ink colors of color words if there is a mismatch between the ink color and the word) performed better when a confederate was present in contrast to a condition when he or she was absent (Sharma et al. 2010). In detail, the individual learning scenarios, the presence of social cues like digital entities in a digital environment, prime the perception of presence as well (Liew et al., 2017). It was shown that even non-interactive learning materials like videos or audio messages increase the perception of social presence (Mayer 2014b), thereby increasing learning performance when social cues are included. This improvement of performance is described by two explanations: the arousal explanation and the self-awareness explanation (Neider et al. 2019). According to the first explanation, the presence of others or digital entities elicit arousal, which is used to evaluate the responses to a task (e.g., Zajonc 1965, Cottrell 1972; Liew et al., 2017). According to the second explanation, social presence leads to attention towards self-awareness by questioning the own standard of performance (e.g., Carver and Scheier 1981; Duval and Wicklund 1972). In contrast, there are also studies claiming that social presence can lead to negative performance effects suggesting that moderating variables, such as the task complexity, might influence the effect of social presence (i.e., social inhibition; Zajonc 1965; see also; Belletier et al. 2019; Halfmann et al. 2020).

### Social Representations, Social Schemata, and Collaboration Scripts

Based on early attempts to describe social cognition, Moscovici (1981) proposed a theory on social representations. According to this theory, all social knowledge is internalized as social representations. These social representations are explicitly included in the CASTLE as social schemata in the long-term memory. Social representations or schemata comprise values, ideas, beliefs, and practices that are shared among members of a group or even whole communities. These representations are used to cope with their social world but also to be able to communicate with others within a specific environment, even if this environment is non-social. If environments, such as a new digital learning environment, are unknown, people use two strategies to make it familiar: anchoring and objectification. While anchoring is described as ascribing meaning to new phenomena, such as tools or objects, objectification refers to the process of turning something abstract into something almost concrete. These strategies are used independently from a learners’ type of environment.

In later research, the concept of schemata, described as organized mental structures for certain concepts (Rumelhart 1984; Howard 1987), was combined with the social reorientations theory to describe social schemata (for an overview, see Augoustinos and Innes 1990; Fiske and Dyer 1985). According to Rumelhart, these mental structures serve three different functions: (1) understanding oral or written discourse, (2) learning and remembering new information, and (3) solving problems. In terms of social interactions, the term script is used to define a schema for events (e.g., what a person might expect to do in a given situation). Individual social experiences are called self-schemata, while self-schemata are a sub-group of all social schemata (Markus and Sentis 1982; Fiske and Dyer 1985). Self-schemata are stable self-representations facilitating information processing (Markus 1977).

Theories on social schemata and scripts assume that a person’s understanding of and behavior within a social situation follow internal collaboration or interaction scripts. Based on a dynamic understanding of schema theory (e.g., Schank 1999), the script theory of guidance (Fischer et al. 2013) models such scripts as flexible and adaptable knowledge components about social interaction processes and is assumed to include behavioral activities as well as cognitive processing. For example, an internal script may include activities like critically questioning the contributions of others or constructing arguments for a position (Weinberger et al. 2007). The configuration of script components is influenced and potentially activated not only by personal goals but also by perceived situational characteristics (Fischer et al. 2013). These situational characteristics may also include awareness of the social environment, such as the awareness of others’ opinions in a digital learning material (i.e., group awareness; Schnaubert et al. 2020b). It can be assumed that when learners perceive social cues within digital learning environments, these may activate cognitive processes based on social or interaction scripts, e.g., questioning content or preparing arguments or explanations. Such preemptive activities may be comparable to cognitive activities conducted in anticipation rather than the presence of a social situation as discussed in the literature (e.g., Levine et al. 1993) and may be beneficial for learning. Thus, when activated via perceiving social cues, such scripts may guide a learner’s understanding of and behavior within the environment even if there is no “real” social interaction.

### Social Agency Theory and Social Cues

The social agency theory is in line with the CASA paradigm and the media equation theory (Mayer et al. 2003). According to this theory, not only the interaction between humans but also the interaction between humans and digitally presented learning materials is primarily social. Thus, social cues are included in the CASTLE as stimuli included in the digital material. The social reactions of learners to digitally presented learning materials are not only common but also unavoidable. This is mainly because the interpretation of the multimedia learning environment as a social interaction partner is very easy to generate (Lee and Nass 2003). Social cues, such as changing third person formulation into direct addressing (i.e., “you” and “your”; Mayer 2001), create the perception of a social presence and activate a social response scheme of the learner. According to Phillips et al. (2007), social cue decoding subsumes human abilities such as perceiving body and verbal expressions of emotion, interpreting verbal and nonverbal cues in terms of what other people think, or judging statements of others in terms of a social relationship.

Based on the CASA paradigm, learning-relevant cognitive processes are thus promoted, and learning performance might increase (Nass and Moon 2000). According to Mayer (2001), deep cognitive processes such as selecting, organizing, and integrating relevant verbal and visual information into a coherent mental representation are enhanced because learners not only interpret educational materials as pieces of information but also as situations of social communication. Thus, learners are engaged not just to deal with abstract information but to fully understand the meaning of this social communication. In consequence, social cues trigger processes that lead to the construction of a mental model that is as complete as possible (i.e., enhanced cognitive processing) so that learning is fostered. Furthermore, the implementation of social entities in digitally presented learning materials can increase motivation and positively change attitudes towards the learning material (Lester et al. 1997; Tung and Deng 2006). In sum, digitally presented learning materials are by no means perceived as a de-personalized source of information. Rather, simple social incentives lead to the activation of social and parasocial interaction schemes. These socio-cognitive processes may promote profound cognitive processing of learning content and thus learning performance.

### Feedback and Social Processes

In learning contexts, feedback is defined as all information provided to learners related to their actual state of learning and/or their learning outcome (e.g., Hattie and Timperley 2007; Narciss, 2008) and, thus, affect not only the cognitive processing of information but also a learner’s self-concept. In detail, multimedia learning materials can be designed to give learners feedback on their state of learning through pre-set output algorithms. There are several meta-analyses (e.g., Gielen et al. 2010; Hattie and Timperley 2007; Mory 2004; Narciss and Huth 2004; Shute 2008; Van der Kleij et al. 2015) and reviews (e.g., Adcroft 2011; Carless 2006; Narciss 2020) conveying how such instructor feedback conveyed via digital materials has to be designed, when it should be used, and why it might work or not. According to the CASTLE, even a perceived emotional change of the learner through the processing of information from a digital material can be understood as a type of feedback (i.e., affect-as-cognitive-feedback-account; Ray and Huntsinger 2017). Some digital learning systems are also able to model learners’ psychological states to provide individualized instruction (i.e., intelligent tutoring systems; for an overview, see Ma et al. 2014). Regarding social processes, people become aware of others (i.e., social presence) through recent activities around artifacts such as feedback mechanisms (Crandall et al. 2008). The visibility of the feedback makes it part of a social process between the learners and a digital learning material (Van der Pol et al. 2008). According to Price et al. (2007), the processing of feedback is a social-constructivist process, since learners do not only compare information but also try to understand the social background of the information. Also, the more information is known about the feedback giver, the more learners invest mental effort in processing the feedback. Studies have shown that feedback through media materials like videos establishes a better social presence of the instructor (Aragon, 2003). Instructor immediacy to students increases student confidence and resembles a face-to-face learning experience (Atwater et al. 2017; Borup et al. 2012; Griffiths and Graham 2009). As a result of the inclusion of feedback, students feel more connected to their online learning material (Edouard 2015). This increased parasocial interaction can enhance learning (Hung 2016).

### Conflict Elaboration Theory and Socio-cognitive Conflicts

While feedback is aimed at the learner’s state of learning, cognitive information assigned to other learners or groups can also trigger individual learning processes. For example, information indicating a diverging perspective within a group can be used as evidence about reality (informational social influence; Deutsch and Gerard 1955) and lead to (socio-)cognitive conflict. Piaget’s conception of cognitive conflict views information inconsistent with an individual’s cognitive structures as an initiator for assimilation and accommodation processes (Piaget 1977). However, when the information is assigned to a (digital) social entity, the perceived conflict may have a social as well as a cognitive component and thus be socio-cognitive in nature (Butera et al. 2010), which may trigger various forms of conflict regulation (e.g., epistemic, competitive-relational, protective-relational; Butera et al. 2019) and learning-relevant processes according to the CASTLE. The conflict elaboration theory proposed by Mugny and colleagues (Mugny et al. 1995) aims at integrating research on social influence from various traditions focusing on conflicts emerging during an interaction. Based on findings that cognitive performance cannot entirely be predicted by the cognitive performance of group members within groups, Mugny and Doise (1978) extended ideas of imitation in social contexts by proposing that social interaction processes can trigger knowledge construction processes through experiencing and regulating socio-cognitive conflict even in digital learning environments. Such conflict may thus trigger reorganization and restructuring of cognitions (Bell et al. 1985) and epistemic curiosity (Berlyne 1960). This can result in deep processing to integrate the views of others and ultimately promote learning (Buchs et al. 2004). While the type of conflict experience may severely influence the regulatory processes triggered to resolve the conflict (Mugny et al. 1995), the potential of being confronted with diverging perspectives is a key component of group learning scenarios (Johnson and Johnson 2009), but may not be limited to collaborative practices. Although often avoided in individual educational settings, conflicts have been shown to trigger beneficial information processing (Doise et al. 1998; see also Levine et al. 1993) and may also be perceived in digital learning environments without the actual presence of other people. For example, Darnon and colleagues (Darnon et al. 2007) showed that the wording of a textual message assigned to a learning partner meant to trigger epistemic versus competitive-relational conflict regulation significantly affected learning outcomes even without an actual partner present. Additionally, perceiving socio-cognitive conflicts may guide individual information searching processes and may lead to uncertainty within individuals without any actual social interaction processes involved (Schnaubert and Bodemer 2016). Social cues within digital learning environments indicating different perspectives on learning content may thereby prompt metacognitive re-evaluation processes and trigger distinct forms of conflict regulation within individuals (i.e., epistemic, competitive-relational, protective-relational).

### Group Awareness Processes in Digital Environments

Social comparison processes and identifying diverging perspectives rely on learners being aware of their (social) environment. Being acutely aware of relevant characteristics of the social environment constitutes group awareness (Gross et al. 2005; Bodemer et al. 2018). The concept originated within the field of computer-supported work (e.g., Dourish and Bellotti 1992) has since been applied to computer-supported learning as well (e.g., Ogata and Yano 2000; see also Bodemer et al. 2018). While targeting a social context (or a context perceived as social), group awareness is an inherently individual concept and includes cognitions about the real or assumed social environment. Group awareness is assumed to emerge in social situations by processing social cues or behavior and communication within (digital) learning environments but also by retrieving information about a social group or specific other from partner models stored in long-term memory (Bodemer et al. 2018). Within digital environments, in which there are reduced communication channels and social context information may be missing, incomplete, or distorted (e.g., Kiesler et al. 1984), a lack of group awareness due to insufficient social cues may severely compromise learning (e.g., Gutwin and Greenberg 2002). Framed differently, social cues within the digital environment may support a specific interpretation of the situation by activating partner models.

While an overarching theory of group awareness is still missing, there is a large body of empirical research investigating the role of awareness about socio-behavioral, socio-emotional, socio-motivational, and socio-cognitive characteristics of the social situation and their impact on cognitive processing and behavioral interactions with digital environments (see Janssen and Bodemer 2013; Bodemer et al. 2018). To support group awareness within digital environments, research focusses on the design and empirical evaluation of specific tools to account for the lack of social cues within digital working or learning environments (Bodemer et al. 2018) and, going beyond that, may provide users with information not directly available in face-to-face interaction, such as socio-cognitive information (see Buder 2011). However, group awareness—as an individual concept—not only affects interaction processes but may influence individual learning as well.

Perceiving (characteristics of) others may make learners change perspectives on the learning content or own learning processes. For example, learners perceiving others having questions may lead them to doubt their own abilities, possibly due to re-evaluations of task difficulty (Karabenick 1996). Since the effects of social cues within digital learning environments inherently rely on their perception and processing by learners (i.e., their awareness), research on the selection and arrangement of such cues is highly relevant for theories on social processes in digital and social environments.

While group awareness research decisively looks into how learners perceive their social environment, the perception of said environment may also affect how learners position themselves within the social context.

### Social Identity Theory

The social identity theory explains that parts of people’s self-concept are constructed through interactions with real social groups (Tajfel 1978) but, in line with the CASTLE, also with digital entities (Edwards et al. 2019). By this, learners can categorize new information in social interactions into (para-)social groups. These social groups differ in their value and emotional significance. For example, when new information is similar to the concept of a social group that is high in value and emotional significance, more attraction, and social identification is triggered.

Studies have shown that the concept of social identification can also be transferred to individual learning situations (e.g., Edwards and Harwood 2003; Rocca and McCroskey 1999). In a study by Edwards et al. (2019), students with a high age identification motive rated an artificial intelligence system as more credible, higher in social presence compared to students with a low identification. These students also reported putting more motivation into learning with the system. In conclusion, the social identity theory can explain why similarities between a digital learning material and a learner can be social cues that trigger social presence and motivation processes.

To underline the importance of social cues in various digital learning environments, the following sections include empirical results emphasizing the importance of social processes postulated in the CASTLE. Since these processes may vary within different sources of information, these sections are separated according to (1) the modality of information (verbal vs. visual), (2) the dynamics of the information presentation (static vs. dynamic), and (3) the interactivity of the environment (interactive vs. non-interactive).

## Social Cues in Static Verbal Learning Materials

### The Personalization Effect

One way of activating social schemata in learners is described by the personalization effect. This effect is based on social agency theory and was described by Moreno and Mayer (2000) as a way to improve learning outcomes when using colloquial expressions in texts compared to formal expressions. In a learning environment by Mayer et al. (2004), for example, direct articles were exchanged with the possessive pronoun “you/yours” to achieve higher learning performance. But the personalization effect can also be applied in instructions. Thus, in a learning material with direct addresses of learners, prompts achieved higher learning outcomes than general instructions (Moreno and Mayer 2004). The effect can be explained by the activation of self-schemata (i.e., one type of a social schema), increased familiarity, as well as a rise in interest and relevance for learners (e.g., Reichelt et al. 2014, Schneider et al. 2015a). The effect of personalization has now been proven by a large number of studies (for an overview, see Ginns et al. 2013).

However, the personalization effect is subject to different moderators visible through empirical findings of various studies. On the one hand, learners with low prior knowledge benefit more from personalized materials than learners with a high level of prior knowledge (e.g., Stiller and Jedlicka 2010). On the other hand, the type of learning content (e.g., social science vs. natural science) and the type of learning time limitation (self-directed vs. externally controlled) can be decisive for the effectiveness of this design principle (Ginns et al. 2013). Furthermore, the effectiveness of personalization can also vary from country to country and from language to language, because social cues appear in different forms in different cultures (e.g., Brom et al. 2017). Despite these limitations, the personalization effect represents an easy way to activate social processes through digital learning materials. However, experimental verification concerning these factors is still necessary to show the limits of the effect.

### Cultural Cues as Social Cues

Apart from the personalization effect, which is influenced by cultural differences, other design decisions only occur in differences between cultures or only become apparent within a specific cultural group. These culturally influenced cues are called “cultural cues” (Schneider et al. 2015b). An example of a culturally influenced effect is the politeness effect. This effect describes that learners learn better with polite instructions (e.g., Mikheeva et al. 2019; Schneider et al. 2015b) or polite feedback (e.g., McLaren et al. 2011a, 2011b) rather than with direct language forms. The effectiveness of polite feedback is explained by the politeness theory of Brown and Levinson (1987), which states that people strive to save face in interaction (even with digital environments). The theory is based on the socio-psychological foundations of communication according to Goffman (1974) and Grice (1975). All influences of an environment on a learner should, therefore, be designed in such a way that the requirements of learners are recognized and that they are restricted as little as possible in their perceived autonomy or freedom of action. This can motivate learners to continue to engage with the learning environment and achieve higher learning outcomes (Wang et al. 2008). However, this effect is strongly limited to cultures that differentiate a form of politeness from, for example, a form of direct speech (e.g., within the Turkish language; Kurt 2011).

Similarly, this is true for language forms that include a limited geographical area (dialects) or are spoken in a limited social group (sociolects). In a study by Rey and Steib (2013), the use of a native dialect at an Austrian school led to a higher situational interest in the learning environment and better learning performance compared to a standard German variant. The use of youth language as a sociolect in a study by Schneider et al. (2015a) also showed increased learning performance compared to a learning environment designed in a standard German language. In both studies that used one language form, the results were attributed to social effects during learning. In summary, cultural signs can be understood as a “variety” of social processes in learning materials. While social cues are widely considered to be researched, cultural cues on different social phenomena are largely unexplored.

## Social Cues in Static Visual Learning Materials

### Emotional Design as Social Cues

Social situations do not arise exclusively through the use of socially shaped language forms, but can also be reinforced or weakened by the emotional design of a learning environment or by dealing with the emotional information in a learning environment (Muller 2004). The emotional reaction to learning material is also interpreted by the learner as social information because the learner is given information about the current situation or the intention of the author of the material (emotion as social information; Van Kleef 2009). Pekrun and Stephens (2010) define this class of socially related emotions as social emotions. Even in digitally presented learning materials where the social presence and visibility of other people is reduced, social emotions are communicated (Derks et al. 2008). This can also be transferred to learning media and has led to an increased investigation of social processes triggered by emotions in recent years. Thus, positive emotions have been specifically evoked or varied by design elements in learning materials (e.g., Plass et al. 2014). In a study by Schneider et al. (2016), positively emotional images have a learning effect compared to negative images and increase the satisfaction of the learners. This effect was replicated in another study by Schneider et al. (2018a). The choice of a color concept can also affect the emotional state of learners and increase motivation (Heidig et al. 2015). By triggering emotions in learning materials, some social information is always provided (e.g., the last social situation in which this emotion occurred). There is also research that examines the design of digital learning materials as a possibility to affect learners’ emotions by altering colors or the degree of anthropomorphism in order to foster learning (for an overview, see Brom et al. 2018; Wong and Adesope 2020). However, the influence of emotions on social influences when learning with media has hardly been investigated so far and should, therefore, be the subject of future research.

### Human Representations and Anthropomorphism

An additional possibility to trigger social processes in digital learning materials is the human representation or human-like forms. According to the theory of anthropomorphization, this design principle is automatically linked with a social response. Anthropomorphization is the mental transfer of human characteristics, motivations, intentions, and emotions to non-human living beings or artifacts (Epley et al. 2007). For example, a learning environment that contains a human-like agent is perceived by the recipient as anthropomorphic and thus is provided with human characteristics (Epley et al. 2007). Anthropomorphization thus joins the research on the CASA paradigm. The basal cognitive operations triggered by anthropomorphization influence both the information selection, the activation of already learned knowledge, and the application of this knowledge to new objects (Higgins 1996). Anthropomorphization processes take place already in pre-attentive or pre-cognitive phases and are triggered by shape, movement, and noise information (Lemaignan et al. 2014). When human figures appear in a learning environment, influences such as morphology or ethnicity play an important role to serve as triggers for anthropomorphization (Waytz et al. 2013). Other triggers may be human-like human voices (Lee 2010) or a phylogenetic similarity of the image to humans (Eddy et al. 1993). Especially in the interaction with digital technologies, a variety of anthropomorphization mechanisms occur as the users of the technology try to make technical processes understandable for themselves (Persson et al. 2000). Anthropomorphization can lead to a variety of phenomena. For example, a new environment can increase the level of care, trust, and responsibility, as well as the degree of allowed influence (Waytz et al. 2010). This also increases the perception of social interaction with the learning material (Gong 2008). On the motivational side, the degree of anthropomorphization also increases intrinsic motivation in the form of a tendency to process information more closely (Epley et al. 2007). This, in turn, leads to a higher learning performance (e.g., Blanchard and Mcnincth 1984; Park et al. 2015; Schneider et al., 2018b; for a meta-analysis, see Brom et al. 2018). The degree of anthropomorphization thus has a decisive influence on the perception of learning materials as social interaction partners.

In the context of anthropomorphization, several design options for digital learning materials were investigated. For example, the display of human facial features in non-human images (e.g., Schneider et al., 2018b) or the presentation of entire persons (e.g., Frechette and Moreno 2010) triggers such anthropomorphization processes. One example of such presentation can be found in the inclusion of pedagogical agents (see section “The Special Case of Pedagogical Agents”). However, there might be learning differences when examining different age groups or graduation of anthropomorphization (e.g., low, medium, or high; Schneider et al. 2019). However, it is still unexplored to what extent new forms contribute to triggering anthropomorphization (e.g., animal characteristics) and support or inhibit the learning process.

## Social Cues in Dynamic Learning Materials

### Social Processes by Interacting with Dynamic Learning Materials

Recent investigations pointed out that stronger (para-)social processes lead to higher learning outcomes when learning with instructional videos (Beege et al. 2017a; Beege et al. 2019). According to the two-level model of parasocial interaction (Hartmann et al. 2004), two explanations can be derived.

At first, the cognitive-perceptive facet of PSI is important. An enhanced PSI is associated with cognitive processes such as attention, understanding, and evaluation (Hartmann et al. 2004). For example, social cues like gestures of a digital social entity draw the attention of the learner directly to the lecturer (and therefore, directly to the speech; see Wakefield et al. 2018) and foster learning processes (embodiment principle; Glenberg 2010; Mayer 2014a). Beege et al. (2017b) showed that a persona’s eye contact with a recipient led to increased PSI and improved learning outcomes. Thus, even simple cues seem to be a very effective form of social encounter that fosters parasocial processes like attention, engagement, and evaluation processes and, therefore, learning.

Another explanation can be derived from the affective facet of PSI. Gola et al. (2013) pointed out that children’s learning performance is increased from unknown personae over time because children form emotional bonds with these figures. Calvert et al. (2014) extended these findings by stating that 18-month-old children learn better with personalized personae. For example, personalization was operationalized through favorites (e.g., the same food or song preferences). Another approach for learning benefits of affective parasocial processes was discussed by Brownlow (2014, 2015). Brownlow stated that PSI fosters learning through emotional processes of empathy and antipathy towards social entities. An induced sympathy towards the persona leads to reflection of the own behavior and approximation to the behavior of the persona. In consequence, the affective facet of PSI acts as a stimulus for change.

### Social Cues in Auditory Messages

According to the voice principle, spoken sentences should be pronounced with normal intonation and not with a mechanically distorted voice or a foreign language accent. It is explained analogously to the personalization principle (Mayer 2014b) and supported by several experiments (e.g., Liew et al. 2020; Chiou et al. 2020). Mayer et al. (2003), for example, conducted two experiments on lightning showing that an audio commentary presented in a conventional accent instead of a foreign language accent (experiment 1) or a machine voice (experiment 2) improved the transfer learning performance and led to more positive evaluations of the speaker. Recent studies outlined that modern speech synthesis software can generate adequate speech quality to credibly and effectively deliver verbal information (Craig and Schroeder 2017, 2019), which may also result in more reliable social cues in digital learning environments.

Nevertheless, the voice principle is more complex than just using human voices instead of computer-generated voices. The prosody of the voice affects social perception as well as learning outcomes, in particular with regard to non-native speakers (Davis et al. 2019). Audio tracks provide social cues (Nass and Gong 2000) in terms of the affective state and personality of speakers, their social and racial group membership, as well as their gender (for an overview, see Edwards et al. 2019). However, in an experiment by Ahn and Moore (2011) manipulating the instructor accent (native vs. European vs. Asian accent), no significant perception difference was found, but it was shown that the students’ perception of accents influenced their learning performance. Students who had previously expressed a lower preference for Asian accents showed poorer learning performance with corresponding speakers than students who preferred this accent to a greater extent. Besides cultural differences, previous studies showed a speakers’ enthusiastic voice leads to higher social and affective ratings and an increased transfer performance compared to a calm voice (Liew et al. 2020). Also, a high-quality voice (made with a highly elaborated speech engine) leads to higher ratings of credibility and engagement, compared to a low-quality voice (Chiou et al. 2020). Recent research further outlines the importance of the quality of the voice on perceived trust in the online learning environment. A high-quality voice increases the perception of trust (Craig et al. 2019). Nevertheless, perceived trust does not seem to have strong influences on learning outcomes (Schroeder et al. 2021).

However, the vast majority of possible voice factors, such a tone, pitch, intonation, and others, are not yet fully examined in terms of eliciting social processes. Further research in this area with larger samples, cultural comparisons, and moderating factors, especially in connection with actual speech synthesis programs, is therefore urgently needed.

### Movements as Trigger for Social Cues

Based on the theory of anthropomorphization as well as the CASA paradigm, not only static information can be perceived as a social cue but also dynamic information (Feine et al. 2019). A broad theoretical and empirical basis was outlined concerning the use of gestures in multimedia learning environments. Gestures draw the attention of the learner directly to the lecturer (and therefore, directly to the speech; see Wakefield et al. 2018). Watching hand movements activates the motoric system of the learner and supports the construction of mental models. This could be shown in studies with actual humans as lectures (e.g., Ping et al. 2014) as well as digital (e.g., Wang et al. 2018). Gestures are particularly conducive to learning when important visual information is highlighted through deictic movements (Li et al. 2019). Deictic gestures help learners to identify which piece of information is necessary for understanding and to direct attention to this information. Again, this assumption was supported by research about actual lecturers in video lessons (e.g., Pi et al. 2020) as well as digital agents (Dunsworth and Atkinson 2007; Davis 2018). Thereby, it is important that the gestures point directly to the information (Craig et al. 2015) and exactly at the time (or only slightly after) the information is presented (Twyford and Craig 2013). In contrast, non-specific, conversational gestures can even inhibit learning (Moon and Ryu 2020).

In dynamic media, the attention direction of the lecturer can not only be guided through gestures; implementing hypothetical eye movements can be beneficial as well. Showing eye movements of an expert during learning with dynamic visualizations supports the attention allocation of the learner (Jarodzka et al. 2013; Krebs et al. 2019). Another way how virtual lecturers can interact with dynamic visualizations is by creating them live during the lecture. Drawing information live instead of relating to already finalized and displayed information increases learners’ comprehension of the material (Lubrick et al. 2019; Fiorella et al. 2019). Viewing a lecturer that dynamically generates relevant information establishes a kind of social partnership between the learner and the instructor which, in turn, fosters generative processing of the material (Fiorella and Mayer 2016). In addition to targeted movements in dynamic learning media, other, more basic, dynamic cues influence the perception and information processing of the learner. For example, Morewedge et al. (2007) pointed out that the speed of movement influences the perception of, for example, intention, consciousness, and intentions of humans and non-human entities like robots. An extensive taxonomy of Feine et al. (2019) further outlined the importance of head movements, facial expressions, and posture shifts. Summarized under the category “kinesics,” these movements from social entities can improve, for example, collaboration and cooperativeness with social entities (Cassell 2000), believability and persuasion (De Rosis et al. 2003), as well as perceived trust and system skills (Cassell 2001).

### The Special Case of Pedagogical Agents

Besides the effect of actual humans in dynamic learning environments, a lot of research was done regarding pedagogical agents. Pedagogical agents are computer-generated or designed characters that serve instructional purposes in educational settings (Veletsianos and Russell 2013; Martha and Santoso 2019). According to Schroeder and Gotch (2015), pedagogical agents can demonstrate tasks, can coach the learner during knowledge acquisition, can be an information source, and/or can act as a test administrator. Thus, there are various potential influences of agents on learning. Overall, a meta-analytic review found that pedagogical agents produced a small significant effect on learning (Schroeder et al. 2013). Nevertheless, the underlying mechanisms of this effect are not clear to date. Heidig and Clarebout (2011) postulated that conditions in which agents are effective and design features should be the focus of research. In general, various effects were outlined by Schroeder and Gotch (2015, p. 184) postulating possible effects on whether digital agents should be seen at all. Even if on-screen agents seem to be more effective than off-screen agents (Schroeder et al. 2013), the visual design is still under investigation. Besides general design features (see the previous section “Human Representations and Anthropomorphism”), researchers focused on, for example, perceived professionalism (Kim and Baylor 2016), perceived sex appeal (Wang and Yeh 2013), demographic features (Baylor and Kim 2004; Beege et al. 2017a), and visual stereotypes (Veletsianos 2010). Besides the visual appearance, voice and movement/nonverbal characteristics were under investigation. General effects which were outlined in the previous two sections are adaptable to the context of digital agents. More specifically, researchers investigated how the speech of a pedagogical agent can act as a cue for perceived verbal competency (Kim et al. 2006), verbal motivation (Domagk, 2010), or the instructional role (Baylor and Kim 2005). Furthermore, nonverbal cues and the degree of embodiment are investigated to determine if the movement of an agent may induce an irrelevant load or whether it helps learners to understand the content (Baylor and Kim 2009; Lusk and Atkinson 2007). When learners are also able to interact (i.e., get feedback to an input to the system), pedagogical agents can also be defined as *interactive* which led to a new body of research on intelligent tutoring systems (for reviews on the effect of such systems on learning, see Graesser et al. 2018; VanLehn 2011). Thus, the effects of social cues elicited by such an interactive form of media need to be further discussed.

## Social Cues in Interactive Learning Materials

### Social Cues Elicited Through Social Comparison

Interactive media can be used to provide multifaceted social cues influencing learning, ranging from binary information (e.g., someone is better/worse), over discrepancies (someone can calculate *twice* as fast), via conflicting assumptions (someone proposes a different solution), to complex schemata and scripts (e.g., the understanding how others solve a mathematical problem). Following this continuum, different processes can be observed. Simplistic social information (e.g., a numerical representation of a rank) triggers comparison processes and results in competitive behavior (Nebel et al. 2016a; Nebel et al. 2017a). As a consequence, behavioral goals are set and pursued. Depending on their nature (e.g., distant vs. close; Locke and Latham 2002; specific vs. unspecific; Nebel et al. 2017b), learning is influenced differently. Further effects might be induced depending on whether the competitive goal is aligned with the underlying learning goal, especially as learners might struggle to transfer from competitive to learning behavior (Chen 2014) or no deep learning is triggered (DeLeeuw and Mayer 2011). Individual or cultural differences (e.g., different prior knowledge; ter Vrugte et al. 2015; competitive anxiety; Hong et al. 2015; cultural norms; Gibbons and Buunk 1999) alter the outcomes of learning processes even further. If the learning material includes more complex social cues (e.g., virtual representations of the competitors and their actions), extraneous cognitive load could be induced as well (Nebel et al. 2016b), although this may depend on how social data is presented (Schnaubert et al. 2020a).

### Social Cues Through Feedback

Feedback can be seen as a social cue triggering social comparison processes and also have an impact on, for example, self-awareness and self-schemata (see above). According to Lin and colleagues (Lin et al. 2013), especially elaborated feedback let learners experience digital agents as a more authentic and credible learning companion. This activates learners’ social schema and promotes an interaction with the computer environment and learning process. In addition to the meta-analytically proven positive effects of feedback (e.g., Van der Kleij et al. 2015), negative effects of feedback can also occur (e.g., Fyfe and Rittle-Johnson 2016; Kluger and DeNisi 1996). For example, feedback on a (poor) learning performance can distract attention from the learning task and lead to irrelevant thinking, thoughts about other people, or a comparison of learning performance with other learners. In this regard, Clark-Gordon et al. (2018) point out that learners often view feedback as a face-threatening event. In two studies, they were able to demonstrate that verbal and nonverbal face-threat mitigation strategies can counteract this (Clark-Gordon et al. 2018). Overall, feedback triggers a social reaction that can lead to changes in the processing of information. Moreover, feedback as a social cue can trigger emotional, motivational, and metacognitive processes (e.g., Naismith and Lajoie 2018; Ray and Huntsinger 2017; Tricomi and DePasque 2016).

### Complex Social Cues

Gathered information beyond binary or simply quantifiable information triggers more complex behavior as this evolves beyond Festinger’s (1954) postulated comparison of abilities. Within collaborative learning research, perceiving discrepancies and experiencing conflict is assumed to be a driving force of individual and collaborative learning (see Schnaubert et al. 2019; Schnaubert and Bodemer 2019). While traditionally this line of research focuses on social interaction processes, research in the context of self-regulated and computer-supported collaborative learning indicates that conflicts or controversies may also affect learning processes beyond social interaction. Depending on the setting and the information perceived, information on others has been found to trigger adjustments of own metacognitive experiences within various fields of research (e.g., McGarty et al. 1993; Koriat et al. 2018), leading to adjustments in study regulation (e.g., Schnaubert and Bodemer 2016, 2019). For example, in a series of experiments, Schnaubert and Bodemer (2016, 2017, 2018, 2019) studied how providing individual and collaborating learners with socio-cognitive and (socio-)metacognitive information during learning affect learning regulation, finding strong guidance effects on topic selection and study time regulation, but inconsistent effects on cognitive and metacognitive learning outcomes. Other studies also found evidence of visual information on conflicts guiding individual learning processes, for example, in a wiki environment (Heimbuch and Bodemer 2017), or collaborative learning processes, for example, with a collaborative multimedia application for statistics (Bodemer 2011). Indications that conflict awareness may be particularly beneficial for the integration of different perspectives or types of information (e.g., Bodemer 2011; Heimbuch and Bodemer 2017). However, it is assumed that these effects strongly depend on how social cues are processed and arranged to foster inter-individual comparison processes to help learners to identify conflicting assumptions and suggest beneficial conflict resolution processes (see Schnaubert et al. 2020a).

Apart from conflicting information and cognitive re-evaluation processes, social cues may not only focus learners’ attention but help them model favorable behavior by imitation. Perceived social cues about other learners’ behavioral patterns and/or recommendations within digital social learning environments can potentially foster indirect or direct social navigation (see Buder and Bodemer 2008). Social navigation systems in a broad sense incorporate recommender systems as well as other forms of awareness information gained by tracking behavioral patterns of users (Dourish 1999). Within digital and social learning environments, awareness about other learners’ recommendations has repeatedly been found to trigger such social navigation processes for information visualizing agreement and quality ratings (e.g., Buder et al. 2015) or novelty (Buder and Bodemer 2008).

## Discussion

Previous theories on learning with media such as the CLT (Sweller et al. 1998, 2019) or the CTML (e.g., Mayer 2014a) have primarily highlighted cognitive processes in learning. Theoretical extensions such as the aCLT (Huk and Ludwigs 2009), CATLM (e.g., Moreno 2006), or ICALM (Plass and Kaplan 2015) prove that these cognitive theories have now been supplemented by affective, motivational, and metacognitive processes. However, the role of social processes within digital and multimedia learning environments still lacks an overarching theory. Therefore, this article outlines the Cognitive-Affective-Social Theory of Learning Environments (CASTLE) as an attempt to integrate social processes in a unified theory concerning learning with digital materials.

The theory is theoretically incorporated into several existing approaches to social processes such as media equation theory (Reeves and Nass 1996) and CASA paradigm (Nass et al. 1994), but also due to social presence theory (Short et al. 1976), social identity theory (Tajfel 1978), and PSI theory (Hartmann et al. 2004). Furthermore, the Theory of Social Comparison Processes (Festinger 1954) is especially important as a fundamental framework supporting the inclusion of social comparison processes within virtual (para-)social learning environments.

Besides, previous theories that take up cognitive and social processes are taken into accounts, such as social constructivist theory (Vygotsky 1978), social cognitive theory (Bandura 1986), or socially situated cognition theory (e.g., Smith and Semin 2004). Other theories are also included such as the theory on social representations (Moscovici 1981), the social reorientations theory (for an overview, see Augoustinos and Innes 1990; Fiske and Dyer 1985), the script theory of guidance (Fischer et al. 2013), the social agency theory (Mayer et al. 2003), the conflict elaboration theory (Mugny et al. 1995), or reflections on feedback associated social processes (e.g., Hattie and Timperley 2007; Narciss, 2008).

CASTLE is based on the CATLM (e.g., Moreno 2006) and supplements this approach with a social mediation hypothesis assuming that both social and parasocial processes occur in the reception of digitally presented learning materials and thus influence learning. According to the CASTLE, social cues trigger the activation of social schemes and subsequently lead to (para-)social processes. These processes influence the information selection, further information processing in working memory, as well as the integration and retrievability of mental models concerning long-term memory (see Fig. 1).

**Fig. 2:**
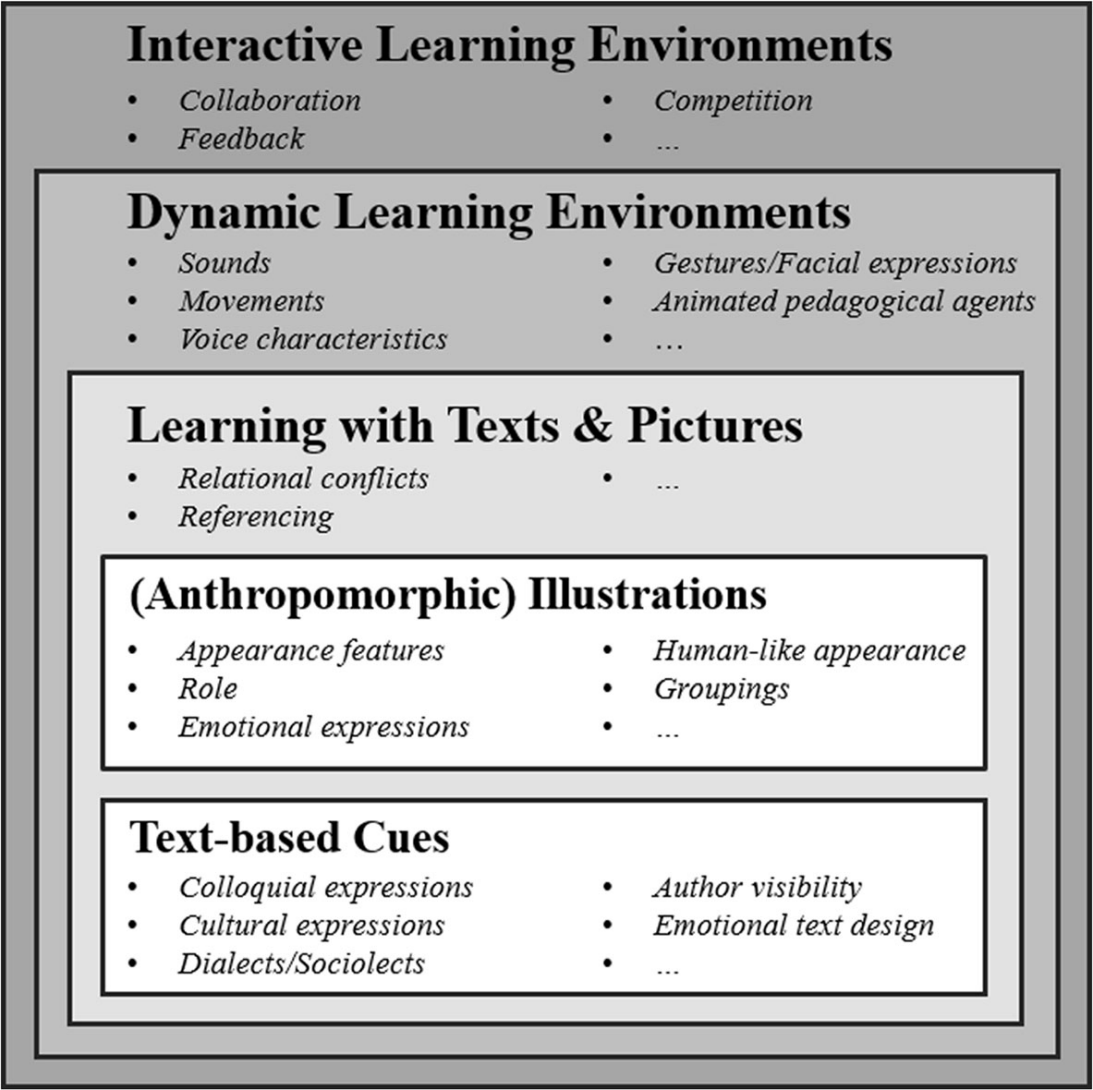
Examples of social cues according to the type of media used in the digital learning environment

The CASTLE can be used to explain postulated effects and empirical findings on social cues in static, dynamic, and interactive learning materials. Figure 2 gives examples of how social cues can be included in different media. The more dynamic and the more interactive a learning medium is, the easier social cues can be implemented since more basic social cues in texts and pictures can also be used in dynamic and interactive learning media. For example, a digital learning environment with pedagogical agents can use both verbal cues and pictorial cues to elicit social processes. If this medium is also dynamic, even more cues can be used. It is also postulated that the number and strength of social cues influence the extent to which social schemes can be activated. Previous empirical findings suggest that with a rising number and higher strength of social cues, the influence of social processes increases. However, effects such as the personalization effect (Ginns et al. 2013) prove that even very simple social cues can lead to social processes being activated in digital learning materials. Further social cues can be found within design recommendations based on the politeness effect, the voice effect, emotional design, anthropomorphization, gestures, pedagogical agents, and “kinesics” as well as social comparisons, collaboration, feedback, and imitation.

In addition to explaining various findings and connecting previously less related research, one main hypothesis and six sub-hypotheses can be derived from the CASTLE and used to guide future research. Subsequently, the creation of learning material can be supported and the knowledge of human learning processes deepened. As argued within the “Introduction,” it is of central importance that the influence of social processes when learning with digital materials should be investigated in closer detail. This paper including the CASTLE and the derived hypothesis can be used as an essential starting point for future empirical research.

### Limitations

Despite the potential of CASTLE concerning the integration of social processes in a unified theory concerning learning with digital materials, this theory also contains several limitations. Like CATLM, CASTLE only allows limited specific predictions of how underlying learning processes work in detail but can rather be viewed as a non-specific framework. Therefore, the numerous social-psychological and cognitive-social theories mentioned in the paper are not fully integrated into CASTLE. Interactions between social processes and (meta-)cognitive, affective, and motivational processes are not further specified in the theory. The model also makes no specific predictions about how and to what extent social cues influence learning performance in detail. Furthermore, CASTLE primarily considers social cues in learning materials rather than real collaboration in groups when learning together with digital materials.

### Future Directions

**Fig. 3:**
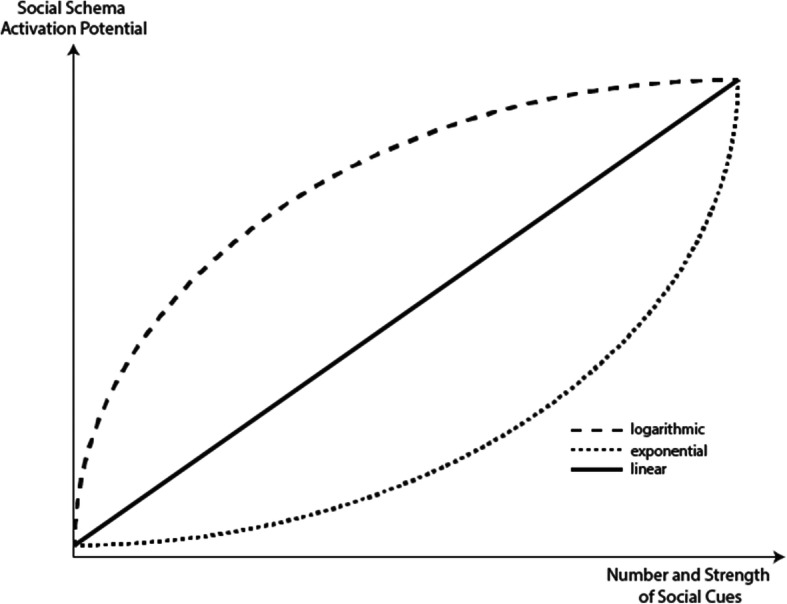
Possible relationships between number and strength of social cues with a social schema activation potential

Future work on the influence of social processes when learning with digital materials should investigate the underlying processes in more detail and examine specific predictions on the influence of social cues on learning. The numerous social-psychological and cognitive-social theories described in the article should be considered for generating specific hypotheses. Future work should, in particular, examine the interactions between social processes and (meta-)cognitive, affective, and motivational processes in more detail and determine their effects on learning processes empirically. However, to analyze the postulated processes, it is vital not only to manipulate social cues experimentally but to look into the perception of said cues by the individual in question. This requires on-line assessment of an individual’s awareness and evaluation of social cues as well as mediation analyses to assess their impact on cognitive, metacognitive, motivational, and emotional processes. Perhaps the connection between the strength and number of social cues and the potential to activate a social schema is crucial in order to explain social processes and their impact on learning. A possible connection between these two concepts is outlined in Fig. 3. If the connection between the degree of social schema activation potential and the number and strength of social cues is linear, quadratic, or exponential, it is still not examined and can be debated in future studies.

By now, there are only a few attempts to examine possible mechanisms using mediation or moderation analyses. While there are single studies on social cues examining the effects of social cues moderated by motivational variables (e.g., Endres et al. 2020; Schrader et al. 2018), emotional processes (e.g., Kühl and Zander 2017; Liew et al., 2017), or cultural variables (Brom et al. 2017), even fewer studies analyzed social processes as mediators of included social cues (Araujo 2018; Gunawardena and Zittle 1997). In particular, it should be fathomed when, how, and to what extent social cues prove to be conducive or a possible hindrance to learning. A similar procedure can be found, for example, with the effects of seductive details in learning materials (Rey 2012; Sundararajan and Adesope 2020) or anthropomorphizations in decorative images (e.g., Schneider et al. 2019). The negative effects from seductive details originally postulated there and the assumed positive effects from anthropomorphizations can be influenced by numerous moderator variables and also lead to reversal effects. Depending on various moderating variables, social cues could also lead to a higher cognitive load, inhibiting individual learning processes in certain situations. Thus, effects like the expertise-reversal-effect (i.e., multimedia effects seem to diminish, disappear, or reverse when prior knowledge is high; e.g., Kalyuga and Sweller 2018) should also be considered when studying the effects of social cues in digital learning environments. In this vein, also age differences might be of importance for the effectiveness of social cues.

Finally, social cues should also be examined in learning groups considering differences and similarities to individual learning scenarios (cf. collaborating working memories; Kirschner et al. 2009). Thereby, the challenges but also potentials of multiple perspectives between individuals interacting with multimedia material, as has been studied in computer-supported collaborative learning research, should be considered (see for example the MUPEMURE Framework; Bodemer et al. 2011) and may complement our view on social processes relevant to learning in digital environments.

In addition, the long-term effects of the inclusion of social cues on learning processes are still not examined. It might be the case that these cues help to better consolidate knowledge in long-term memory because of their connection with already trained social schemata; however, empirical validation of such assumptions is still due. Moreover, the contexts of using social cues in digital might vary tremendously. For example, a computer-supported instruction in times of a pandemic might be even more helpful than times when people can interact in reality. Future meta-analyses should also investigate differences in the effectiveness of different types of social cues. Altogether, insights into the effect mechanisms of social cues may not only help to understand individual learning processes in digital learning environments but also enable targeted approaches to support learning with digital learning material across platforms and domains.
